# Botryococcene Inhibits UV-B-Induced Photoaging by Scavenging Intracellular Reactive Oxygen Species

**DOI:** 10.3390/md24020057

**Published:** 2026-01-30

**Authors:** Hiromi Kurokawa, Makoto M. Watanabe

**Affiliations:** 1 Phycochemy Corporation, 4-19-1 Midorigahara, Tsukuba 300-2646, Ibaraki, Japan; m-watanabe@phycochemy.jp; 2 Faculty of Medicine, University of Tsukuba, 1-1-1 Tennodai, Tsukuba 305-8575, Ibaraki, Japan

**Keywords:** botryococcene, antioxidant, UV-B irradiation, photoaging, skin

## Abstract

Sunlight exposure contributes to human health; however, excessive light exposure to skin, especially ultraviolet B (UV-B), can produce high amounts of reactive oxygen species (ROS) and induce inflammation. Some antioxidants, such as squalene, can prevent UV-B-induced inflammation. C_34_H_58_ botryococcene is the most common triterpene hydrocarbon produced by green alga *Botryococcus braunii*; it is biosynthesized via a pathway similar to squalene and appears to have a similar chemical structure to squalene. However, there are no reports on the bioactivity of botryococcene. In this study, we evaluated that botryococcene can prevent the skin photoaging. Using ESR assay, botryococcene could not scavenge any ROS. However, treatment of epidermis cells with the botryococcene significantly suppressed intracellular ROS production by hydrogen peroxide (H_2_O_2_) and attenuated H_2_O_2_ cytotoxicity. Botryococcene enhanced the antioxidant enzymes in gastric cells, thus botryococcene may scavenge ROS indirectly, not directly. Moreover, botryococcene inhibited production of intracellular interleukin-1 and exhibited suppression of melanogenesis activity by UV-B irradiation. Addition of botryococcene-treated epidermal cells culture medium mitigated the increase in matrix metalloproteinase-1 production and the decrease in type I collagen production induced by UV-B irradiation in dermis cells. These results showed that botryococcene has anti-photoaging effects, including preventing wrinkles and blemishes on the skin.

## 1. Introduction

Sunlight exposure contributes to the prevention of several diseases, such as cancer, hypertension, and diabetes. These effects are attributed to the production of vitamin D, nitric oxide, melatonin, and serotonin [1]. However, excessive light exposure, especially ultraviolet B (UV-B), leads to the production of large amounts of reactive oxygen species (ROS) in the skin, which in turn leads to the production of inflammatory molecules, such as interleukin (IL)-1α and prostaglandin E2 (PGE2), accelerating photoaging [2,3,4,5]. ROS production increases IL-1α and cyclooxygenase-2 levels in epidermal cells. IL-1α enhances tyrosinase activity by mediating increased production of endothelin-1 and stem cell growth factor, thereby enhancing keratinocyte phagocytosis and promoting pigmentation [6,7,8,9]. Additionally, PGE2 production, promoted by cyclooxygenase-2, increases tyrosinase activity [10]. Enhanced IL-1α also promote matrix metalloproteinase-1 (MMP-1) expression, which inhibits type I collagen synthesis, leading to collagen degradation [11,12,13,14]. Therefore, photoprotective agents have been developed to prevent photoaging.

The colonial green alga *Botryococcus braunii* Kützing (Trebouxiophyceae, Chlorophyta) is widespread in freshwater environments and is a well-known producer of hydrocarbons that accumulate in colony matrixes. The hydrocarbon content of this alga is significantly higher than that of other oil-producing microalgae [15], ranging from 20% to 50% *w*/*w* [16]. Various hydrocarbons are produced by different strains of *B. braunii* and are classified into four chemical races based on their chemical structures: A, B, L, [17] and S [15]. Race A is characterized by n-alkadienes and/or n-trienes and their derivatives with odd carbon numbers (C_23_–C_33_). Race B synthesizes specific C_n_H_2n−10_ triterpenes, known as botryococcenes (C_30_–C_37_) and methylated squalenes (C_31_–C_34_), which most frequently occur in natural lakes and reservoirs. Race L synthesizes a single tetraterpenoid hydrocarbon, lycopadiene. Race S produces epoxy-n-alkanes and saturated n-alkane chains with carbon numbers 18 and 20. These hydrocarbons are preferred algal fuels because of their high energy density and availability as materials for chemical engineering. However, the slow growth of *Botryococcus braunii* poses significant challenges to its practical use in biofuel production and as a chemical commodity.

Botryococcenes are biosynthesized via a route similar to that of squalene, which is an essential intermediate metabolite of sterol metabolism in all eukaryotes. Squalene synthase-like (SSL) genes *SSL-1*, *SSL-2*, and *SSL-3* provide triterpene oils with specialized functions in algae [18]. *SSL-1* catalyzes the initial condensation of two farnesyl diphosphate to form pre-squalene diphosphate, which is then reductively rearranged by *SSL-2* to form squalene. Alternatively, *SSL-3* catalytically reduces pre-squalene diphosphate to form botryococcenes through the 1–3′ linkage of the two farnesyl diphosphate molecules.

Squalene is a 30-carbon polyprenyl compound (C_30_H_50_) with six double bonds only in the main axis with an antioxidant nature [19,20] and possesses several bioactivities, including skin-moisturizing, emollient, antioxidant, anticarcinogenic, and anti-inflammatory effects on the human body [19,21,22,23]. Particularly, it quenches singlet oxygen and protects human skin surfaces from lipid peroxidation caused by UV light exposure and other sources of oxidative damage [21]. It is also known to be highly lipid-soluble and has a high number of double bonds, allowing it to easily penetrate the lipid bilayer of cell membranes, and due to this high permeability, it functions as a skin penetration enhancer [24]. So, currently, squalene has many applications, including cosmetics as squalane, a hydrogenated, saturated derivative of squalene [25] and pharmaceutical formulations for disease management and therapy [19]. Many other polyprenyl molecules structurally similar to squalene, such as β-carotene, coenzyme Q10, and vitamins A, E, and K1, perform critical biological functions, give benefits to skin physiology, and thereby are used as component of cosmetic [21].

C_34_H_58_ botryococcene [26,27] (hereafter referred to as botryococcene) used in this study is the most common polyprenyl molecule in race B of *Botryococcus braunii* [15] and contains one double bond in the main axis and five double bonds in the side chain, making it appear similar to squalene. The double bonds in botryococcene are considered reactive points for chemical transformations such as polymerization, hydrosilylation, oxidation, and thiol−ene reactions [28,29,30]. The efficacies of squalene and other polyprenyl compounds, including its antioxidant properties, has been well known and already commercialized [21,31]; however, little is known about the efficacy of botryococcene, despite its similar structure and biosynthetic pathway to squalene.

Given the structural similarity between botryococcene and squalene, and the demonstrated efficacy of SQ on skin, this study investigated whether botryococcene contributes to the suppression of UV-B-induced photoaging in human skin. Using epidermal and dermal cells, we aimed to further understand the function and efficacy of botryococcene on human skin health.

## 2. Results

### 2.1. UV-Vis Spectroscopy of Botryococcene

**Figure 1:**
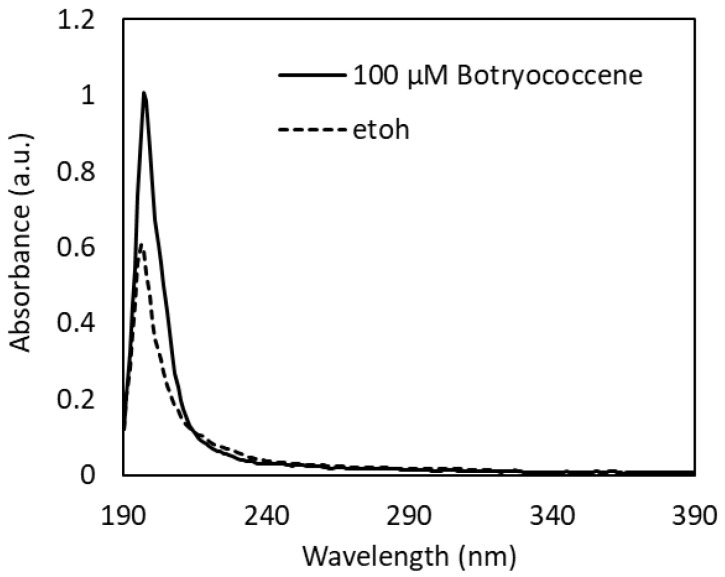
UV-Vis absorption spectra of 100 µM botryococcene dissolved in ethanol.

The UV-Vis absorption spectrum of 100 µM botryococcene dissolved in ethanol revealed an absorption peak at 197 nm in the UV-C region and a little absorption in the UV-B to UV-A region (Figure 1). From the absorption spectrum of ethanol used as a solvent (Figure 1), the small absorption observed in the UV-B to UV-A region is thought to be due to ethanol. Therefore, it can be concluded that botryococcene does not absorb the UV-B irradiated in this study.

### 2.2. ROS Scavenging Ability of Botryococcene

**Figure 2:**
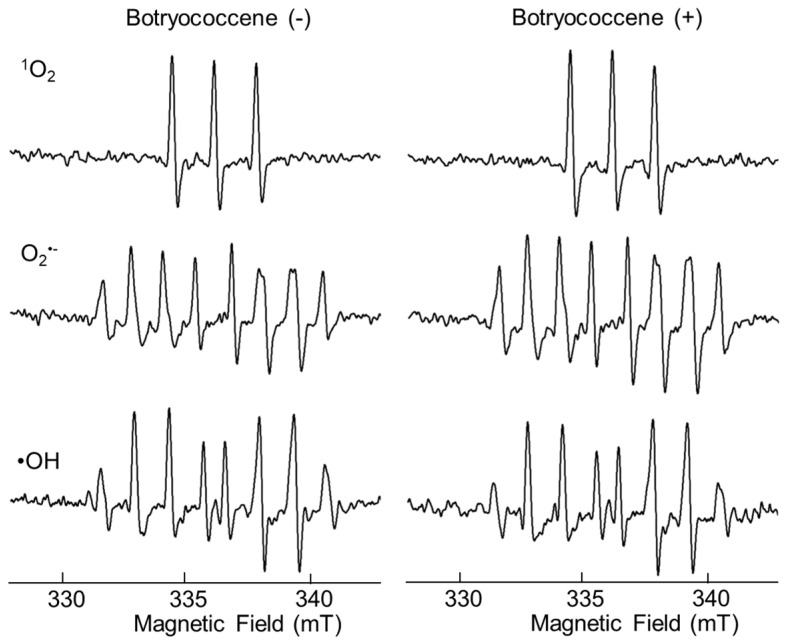
ESR spectra of ^1^O_2_, O2^•−^ and •OH spin with or without botryococcene.

After mixing each radical initiator and botryococcene, the amount of ROS produced in the solutions was measured immediately using ESR. The ESR signal of the mixture containing botryococcene did not differ from that of the mixture without botryococcene, suggesting that botryococcene itself did not have direct antioxidant activity (Figure 2).

### 2.3. Cytotoxicity of Botryococcene

**Figure 3:**
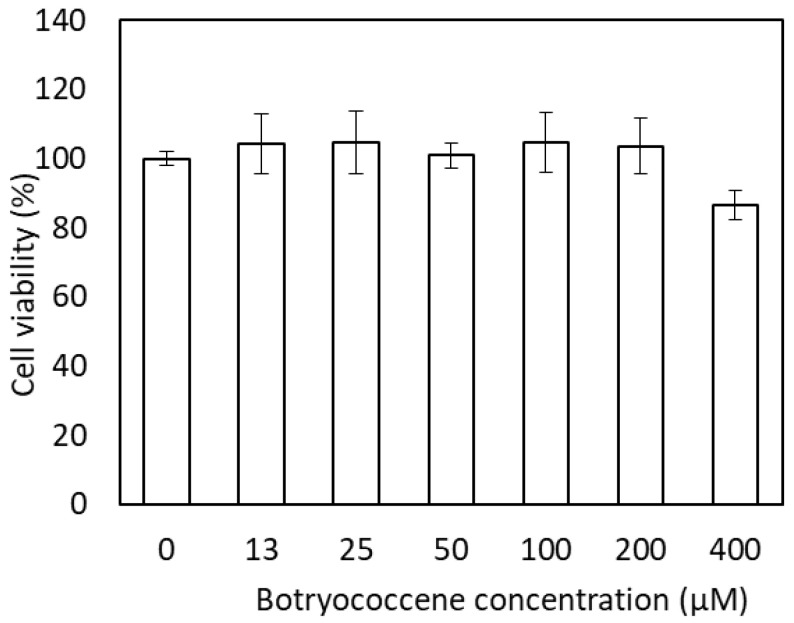
Cytotoxicity of C34H58 botryococcene in NHEKs. Data are expressed as means ± SD (*n* = 3).

The effect of botryococcene on cell viability was measured using neutral red staining (Figure 3). The medium with 0 to 200 µM botryococcene did not induce cytotoxicity. In the medium with 400 µM botryococcene, cell viability decreased to 86.7%; however, a significant reduction in cell viability was not observed when compared with 0 µM botryococcene.

### 2.4. Botryococcene Reduced H_2_O_2_-Induced Intracellular ROS Production

**Figure 4:**
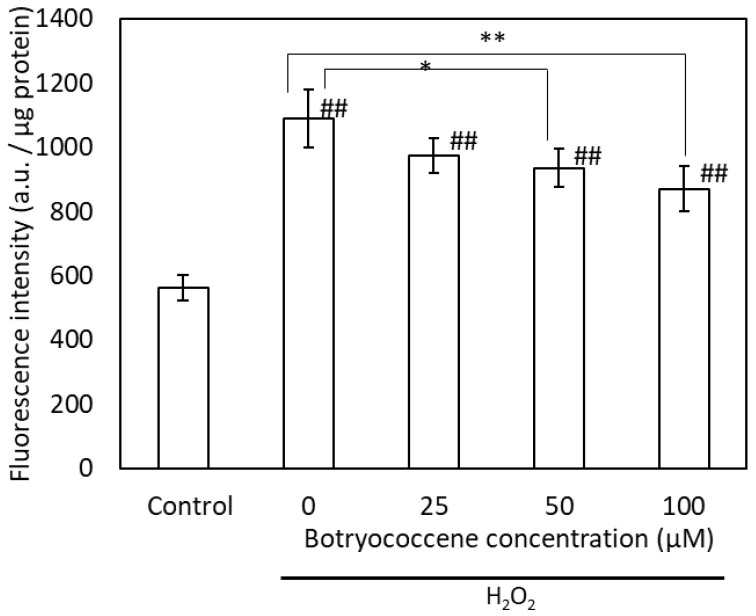
The fluorescence intensity of DCFH-DA after H_2_O_2_ exposure in NHEKs. Data are expressed as means ± SD (*n* = 5). Ex = 485 nm and Em = 530 nm. * *p* < 0.05, ** *p* < 0.01 vs. 0 μM botryococcene. ## *p* < 0.01 vs. Control.

Intracellular ROS production induced by H_2_O_2_ was detected using the fluorescent dye DCFH-DA. High fluorescence intensity indicates high ROS production. The fluorescence intensity of H_2_O_2_-treated cells was higher than that of untreated cells. The intracellular fluorescence intensity of botryococcene-treated cells decreased in a dose-dependent manner following H_2_O_2_ exposure (Figure 4). Particularly, the fluorescence intensity of cells treated with 100 μM botryococcene significantly decreased compared with that of untreated cells. This indicates that botryococcene can reduce the ROS produced by H_2_O_2_.

### 2.5. Botryococcene Attenuated the H_2_O_2_-Cell Injury

**Figure 5:**
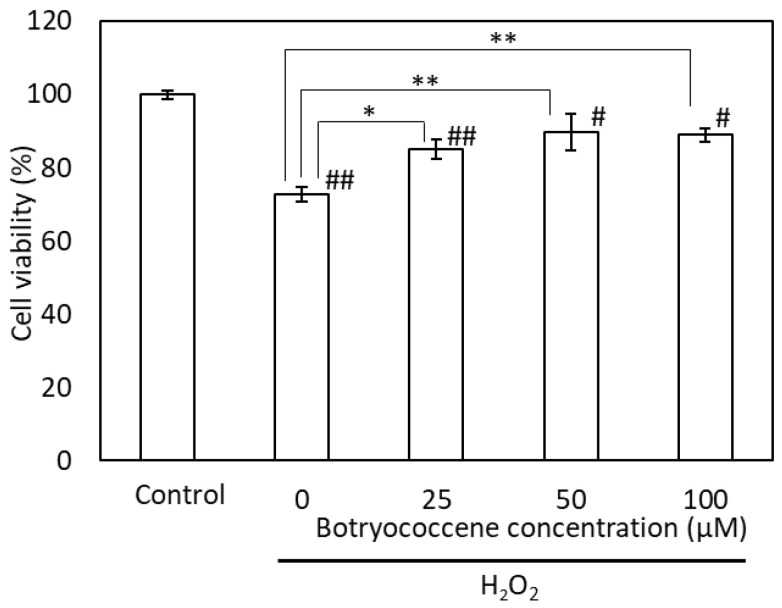
Botryococcene can prevent H_2_O_2_-induced cytotoxicity in NHEKs. Data are expressed as means ± SD (*n* = 4). * *p* < 0.05, ** *p* < 0.01 vs. 0 μM botryococcene. # *p* < 0.05, ## *p* < 0.01 vs. Control.

The cells were incubated with H_2_O_2_ following pretreatment with botryococcene at concentrations of 0, 25, 50, and 100 μM. The viability of H_2_O_2_-treated cells was significantly lower than that of the control and botryococcene-treated cells. Following H_2_O_2_ treatment, the viability of the botryococcene-treated cells increased significantly in a dose-dependent manner (Figure 5).

### 2.6. Effect of Botryococcene on UV-B-Induced IL-1α and PGE2 Generation

**Figure 6:**
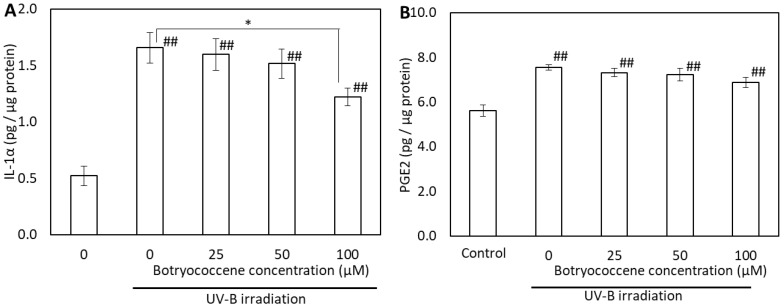
Effect of botryococcene on IL-1α (**A**) and PGE2 (**B**) generation in response to UVB irradiation in NHEKs. Data are expressed as means ± SD (*n* = 4). * *p* < 0.05 vs. 0 μM botryococcene. ## *p* < 0.01 vs. Control.

To evaluate the effects of botryococcene on the generation of UV-B-induced proinflammatory cytokines, the IL-1α and PGE2 in supernatants from botryococcene-treated normal human epidermal keratinocytes (NHEKs) cell cultures were analyzed using enzyme-linked immunosorbent assay (ELISA). IL-1α expression increased after UV-B irradiation (compared with the control); however, it significantly decreased in cells treated with 100 µM botryococcene (compared with untreated cells) (Figure 6A). The PGE2 levels also increased after UV-B irradiation. PGE2 production decreased botryococcene in a dose-dependent manner; however, there was no significant difference compared to 0 µM botryococcene and 100 µM botryococcene (Figure 6B).

### 2.7. Botryococcene Suppressed UV-B-Induced Phagocytosis

**Figure 7:**
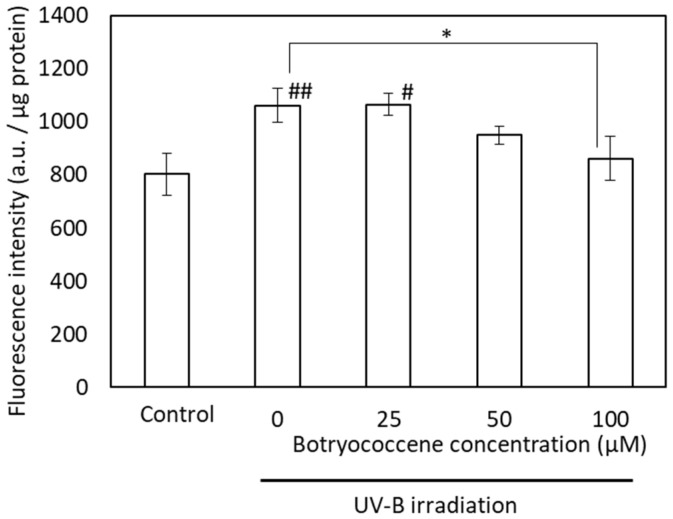
The fluorescence intensity of FluoSpheres after UVB irradiation in NHEKs. Data are expressed as means ± SD (*n* = 5). Ex = 535 nm and Em = 590 nm. * *p* < 0.05 vs. 0 μM botryococcene. # *p* < 0.05, ## *p* < 0.01 vs. Control.

To confirm the inhibitory effect of botryococcene on melanin accumulation, the suppression of melanogenesis activity of botryococcene was measured using FluoSphere beads. Although UV-B irradiation significantly increased the accumulation of FluoSphere beads in response to treatment with 0 and 25 µM botryococcene (compared with the control), the uptake of fluorescence beads upon treatment with 100 µM botryococcene significantly reduced and showed no significant difference with respect to the control (Figure 7).

### 2.8. Botryococcene Treatment Attenuated UV-B-Induced Reduction in Type I Collagen and Elevation of MMP-1 Production in Normal Human Dermal Fibroblasts (NHDFs)

**Figure 8:**
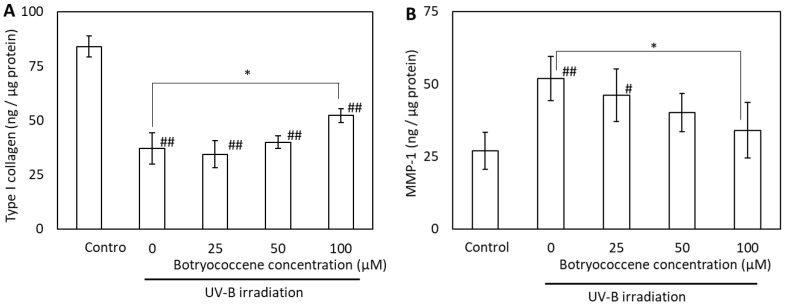
Effect of botryococcene on type I collagen (**A**) and MMP-1 (**B**) generation in response to UV-B irradiation in NDHFs. Data are expressed as means ± SD (*n* = 6). * *p* < 0.05 vs. 0 μM botryococcene. # *p* < 0.05, ## *p* < 0.01 vs. Control.

The effects of UV-B irradiation on type I collagen and MMP-1 production were examined to determine the mechanism by which botryococcene counteracts these effects. UV-B irradiation reduced type I collagen production but induced MMP-1 production in NHDFs (Figure 8A,B). Treatment of NHDFs with 100 µM botryococcene resulted in significantly increased type I collagen production, whereas, inhibited MMP-1 production after UV-B irradiation (compared with untreated NHDFs).

## 3. Discussion

Microalgae have clear advantages in terms of functionality, effectiveness, and safety and reducing greenhouse gas emissions through photosynthesis. Therefore, algae have attracted considerable attention as a next-generation platform for biopharmaceutical production. Important biotechnological applications, including the discovery of novel functional compounds, molecular cloning, genetic engineering, and mass production of algal biomass, have been developed for many species [32].

The green alga *Botryococcus braunii* is widely distributed in lakes, ponds, and dam reservoirs and is known to have a remarkably diverse range of unusual hydrocarbons separated into four chemical races [15,17]. C_34_H_58_ botryococcene which was used and referred to as botryococcene in this study, is the most common molecule produced by many B. braunii strains isolated from natural lakes and reservoirs in Japan [15]. However, little is known regarding the bioactivity of botryococcene. The ethanolic extract of *Botryococcus braunii,* BOT-22, which produces botryococcene, showed antistress- and antidepressant-like effects in a mouse model [33]. However, the components responsible for these effects remain unknown.

The results of this study suggest that botryococcene provides benefits to skin health as an antioxidant, anti-inflammatory, and anti-photoaging agent as shown below.

(1) Antioxidants: The ESR signal showed that botryococcene could not directly remove ROS (Figure 2). However, it does not imply that botryococcene was not involved in the in vivo scavenging process of ROS. The present study using skin cells has suggested that botryococcene is involved in antioxidant processes in vivo. Following H_2_O_2_ exposure, the DCFH-DA-based intracellular fluorescence intensity of botryococcene-treated cells decreased in a dose-dependent manner (Figure 4), whereas cell viability increased in a dose-dependent manner (Figure 5). These in vitro results revealed that botryococcene can scavenge H_2_O_2_ to inhibit H_2_O_2_-induced cytotoxicity, which contradicts the results of the ESR experiments. These seemingly contradictory results can be resolved by considering that botryococcene does not scavenge ROS directly but rather by inducing the expression of antioxidant enzymes in cells, such as superoxide dismutase, catalase, L-cysteine, and glutathione peroxidase. As mentioned in the introduction, botryococcene is a polyprenyl compound structurally and synthetically similar to squalene which has high cell membrane permeability [24]. This suggests that botryococcene can also penetrate into cells. Additionally, in our on-going experiments using gastric cells to observe the effects of botryococcene on the stomach, it was shown that botryococcene acts on the cells and induces the expression of intracellular enzymes (MnSOD, GPx, catalase, etc.) (Figure S1). Both skin cells and gastric cells are epithelial cells, so it is expected that botryococcene treatment will also increase the expression of antioxidant enzymes in skin cells. Therefore, we propose the hypothesis that “botryococcene does not have direct antioxidant activity, but it can acts into cells and induce the expression of intracellular antioxidant enzymes”. Future studies on inducing the expression of antioxidant enzymes by botryococcene using skin cells are required to verify the hypothesis proposed.
(2) Anti-inflammatory: UV-B irradiation increases the levels of inflammatory cytokines, such as IL-1a and PGE2 [3], which is confirmed by this study (Figure 6). Botryococcene decreased the IL-1 production by UV-B irradiation significantly, thus it is suggested that botryococcene can protect the skin damage derived from IL-1 such as inhibition of collagen degradation by Endo180 [34].
(3) Anti-photoaging: Using fluorescent beads, such as pseudomelanosomes, we revealed that the accumulation of fluorescent beads was increased by UV-B irradiation but significantly decreased upon treatment with 100 μM botryococcene (Figure 7), suggesting inhibition of keratinocyte phagocytosis. The present study also revealed that treatment with 100 μM botryococcene increased collagen production and significantly decreased MMP-1 expression (Figure 8). From these results, it is suggested that botryococcene can inhibit skin photoaging [35].

**Figure 9:**
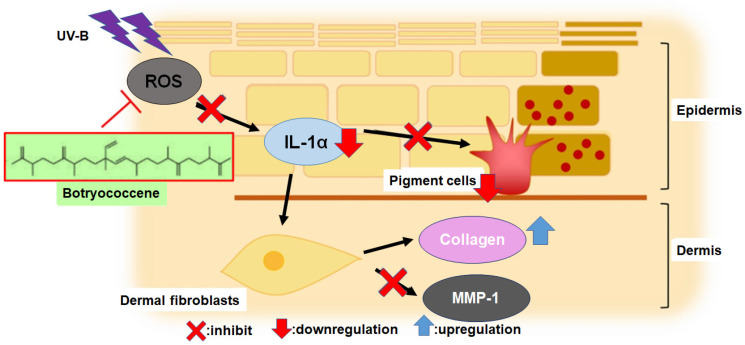
The mechanism of botryococcene-mediated anti-photoaging effect.

The primary mechanisms of UV-B-induced skin photoaging include ROS stress, [4] stimulation of inflammatory compounds, such as IL-1α [3], enhancement of melanogenesis [5], promotion of MMP-1 expression which inhibits type I collagen synthesis, leading to collagen degradation [11,12,13,14]. IL-1α expression, upregulated by UV-B irradiation, activates endothelin-1 and stem cell factor to enhance keratinocyte phagocytosis, [7,8,9] resulting in increased melanogenesis. The present study revealed for the first time that botryococcene does not have UV-B absorption band (Figure 1) and cannot scavenge ROS directly (Figure 2). Therefore, botryococcene acts as a suppressant of each element of the UV-B-induced photoaging mechanism by scavenging ROS via inducing the expression of antioxidant enzymes, reducing the expression of the inflammatory cytokine IL-1α, and inhibiting melanosome accumulation in the epidermis. Particularly, the reduction in the expression of UV-B-induced IL-1α by botryococcene strongly supports its role in inhibiting keratinocyte phagocytosis and preventing epidermal pigmentation of the skin (Figure 9). Moreover, the mechanism underlying photoaging of dermal fibroblasts caused by UV-B irradiation-induced ROS production includes the promotion of MMP-1 expression by the IL family, including IL-1α, IL-1β, IL-6, and IL-8, by activating the mitogen-activated protein kinase signaling pathway, which regulates transcription factors such as activator protein 1 and nuclear factor kappa B, inducing the expression of MMP-1 [36,37,38,39]. Based on the above results, we hypothesize that botryococcene has a photoprotective effect as shown in Figure 9. As a mechanism for suppressing photoaging induced by UV-B irradiation, botryococcene is suggested to reduce IL-1α expression, thereby downregulating MMP-1 expression and inhibiting the decrease in type I collagen.

Since botryococcene has a similar chemical structure and metabolic pathway to squalene, we would like to explain the advantages it has over squalene, which is very commonly used as a cosmetic ingredient. Squalene is an unsaturated hydrocarbon with six double bonds at the main axis. However, it is a very unstable compound because of its easily oxidized nature, and if used as is, its efficacy will be significantly compromised. Therefore, when it is used in cosmetics, it is needed to be transformed into a fully saturated hydrocarbon, called squalane (C_30_H_62_) which has protective effects of ROS and the anti-photoaging effects [25]. On the other hand, botryococcene is not reactive even to ROS, which shows that it is resistant to oxidation. Therefore, there is no need to convert it into saturated hydrocarbons and it can be used as a truly natural cosmetic ingredient.

In conclusion, this study demonstrates that botryococcene can be involved in the suppressing process of UV-B-induced photoaging of skin by scavenging intracellular ROS, probably via the induction of antioxidant enzymes, reducing the expression of IL-1α to alleviate cell toxicity, inhibiting melanosome accumulation, thereby downregulating MMP-1 to promote collagen production.

## 4. Materials and Methods

### 4.1. Chemicals

The sources of chemicals used in this study are as follows: rose Bengal and 4-hydroxy-2,2,6,6-tetramethylpiperidine (HTMP) were purchased from Tokyo Chemical Industry (Tokyo, Japan). H_2_O_2_ and xanthine were purchased from Wako Pure Chemical Industries (Osaka, Japan). 5-(2, 2-dimethyl-1, 3-propoxycyclophosphoryl)-5-methyl-1-pyrroline N-oxide (CYPMPO) was purchased from Radical Research, Inc. (Tokyo, Japan). Xanthine oxidase was purchased from Nacalai Tesque Inc. (Kyoto, Japan). HuMedia-KG2 (KG2) and HuMedia-KB2 (KB2) were purchased from Kurabo (Osaka, Japan). 2′,7′-Dichlorodihydrofluorescin diacetate (DCFH-DA) was purchased from Sigma-Aldrich Japan K.K. (Tokyo, Japan). IL-1α and MMP-1 were purchased from R&D systems (Minneapolis, MN, USA). PGE2 was purchased from Cayman Chemicals (Ann Arbor, MI, USA). BCA Protein Assay Kit and FluoSpheres were purchased from Thermo Fisher Scientific (Waltham, MA, USA). Antibodies against type I collagen were purchased from Rockland Immunochemicals (Limerick, PA, USA).

### 4.2. Botryococcene

Lipids were extracted from the dry biomass of *Botryococcus braunii* Bot-22 under controlled conditions at 25 ± 1 °C using a 2:1 mixture of CHCl_3_/MeOH (*v*/*v*). The extracts were concentrated under reduced pressure, and non-lipid materials were removed by adding one-fifth the volume of a 0.9% NaCl solution (*w*/*w*). After salting out, the CHCl_3_ fraction was concentrated and applied to a silica gel column prepared using CHCl_3_. Four volumes of CHCl_3_ were passed through the column. After evaporation of the eluate, the residue was dissolved in n-hexane. The hexane solution was then applied to a silica gel column prepared with n-hexane, and four bed-volumes of n-hexane were applied to the column. Purity of botryococcene was determined by GC-2030 (Shimadzu corporation, Kyoto, Japan) equipped with a BPX70 capillary column (30 m × 0.25 mm inner diameter; 0.25 µm film thickness). C34H58 botryococcene with a purity of 99% was used in all experiments in this study.

### 4.3. Ultraviolet-Visible Absorption of Botryococcene

Ultraviolet-visible (UV-Vis) absorption spectra of 100 µM botryococcene dissolved in ethanol were measured using a NanoDrop™ 2000 (light source: xenon flash lamp, spectrometer: CCD, optimal path length: 1 mm) (Thermo Fisher Scientific) and recorded from 190 nm (UV-C) to 390 nm (UV-A), including the UV-B region used in this UV exposure study. The UV-Vis absorption spectrum of ethanol, used as a solvent, was also measured and recorded in the same way.

### 4.4. Electron Spin Resonance Assay

The ROS scavenging ability of botryococcene was measured using electron spin resonance (ESR) [40,41]. ^1^O_2_ was generated using a combination of rose Bengal and light irradiation for 60 s (λ = 518 nm). The reaction mixture consisted of MilliQ water containing 200 µM rose Bengal and 10,000 µM HTMP with or without 1000 µM botryococcene. O_2_^•−^ was generated by the xanthine/xanthine oxidase system. The reaction mixture consisted of MilliQ water containing 20,000 µM hypoxanthine, 20 units/mL xanthine oxidase, and CYPMPO, with or without 1000 µM botryococcene. •OH was generated by the combination of H_2_O_2_ and light irradiation for 30 s (λ = 365 nm). The reaction mixture consisted of MilliQ water containing 10,000 µM H_2_O_2_ and CYPMPO with or without 1000 µM botryococcene. The ESR spectra were recorded using a JEOL-TE X-band spectrometer (JEOL, Tokyo, Japan). ESR spectra of botryococcene were obtained under the conditions of 10 mW incident microwave power, 9.4 GHz frequency, 0.2 mT modulation width, 7.5 mT sweep width, 0.3 s time contrast, and 335.5 mT center field.

### 4.5. Cell Culture

NHEKs were purchased from the Kurabo. NHEKs were cultured in KG2 medium supplemented with 10% fetal bovine serum, 1% antibiotics, and 1% human epidermal keratinocyte growth supplement at 37 °C in an atmosphere of 5% CO_2_. NHDFs were purchased from the Kurabo. NHDFs were cultured in Dulbecco’s modified Eagle’s medium supplemented with 10% fetal bovine serum, 1% antibiotics, and 1% human epidermal keratinocyte growth supplement at 37 °C in an atmosphere containing 5% CO_2_. In all the in vitro experiments, botryococcene was dissolved in ethanol.

### 4.6. Cytotoxicity Assay of Botryococcene

To determine the dose range of botryococcene in vitro, cytotoxicity was evaluated using the neutral red method. NHEKs were cultured at a density of 2.5 × 10^4^ cells/well in 96-well plates using KG2 medium for 24 h. The culture supernatant was aspirated and replaced with the KB2 medium containing 0, 12.5, 25, 50, 100, 200, and 400 µM C_34_H_58_ botryococcene, and the cells were incubated for another 24 h. Thereafter, the cells were rinsed with phosphate-buffered saline (PBS) and incubated in KB2 medium containing 0.003% neutral red for 2 h. Following this, the cells were rinsed again with PBS, and intracellular neutral red was extracted using 1 M HCl in 30% MeOH. The A550 was measured using a microplate reader.

### 4.7. Botryococcene Treatment of NHEKs

NHEKs were cultured at a density of 2.5 × 10^4^ cells/well in 96-well plates using KG2 medium for 24 h. The culture supernatant was aspirated and replaced with the KB2 medium containing 0, 25, 50, 100, and 200 µM botryococcene, and the cells were further incubated for 24 h. The botryococcene-treated cells were used to investigating intracellular ROS production, H_2_O_2_-induced cytotoxicity, and UV-B irradiation-induced IL-1α and PGE2 generation; moreover, they were also used for microsphere-based phagocytosis assay.

### 4.8. Intracellular ROS Production

The botryococcene-treated cells were rinsed with PBS and incubated in Hanks’ balanced salts solution (HBSS) buffer containing 200 µM H_2_O_2_ for 1 h. The experimental method was based on the following reference [42]. The supernatant was removed, and cells were incubated in HBSS containing 20 µM DCFH-DA for 30 min. Excitation and emission wavelengths were 485 and 530 nm, respectively. Fluorescence intensity was measured using a microplate reader. The cells were then lysed in 50 μL of PBS containing 0.5% Triton X-100 and transferred to a 96-well plate. The BCA assay was used to estimate the total cell protein.

### 4.9. Effect of Botryococcene on H_2_O_2_-Induced Cell Toxicity

Botryococcene-treated cells were rinsed with PBS and incubated in HBSS containing 300 µM H_2_O_2_ for 1 h. The experimental method was based on the following reference [42,43]. The cells were again incubated in KB2 medium for 24 h. Subsequently, they were rinsed with PBS and incubated in KB2 medium containing 0.003% neutral red for 2 h. Thereafter, the cells were rinsed with PBS. Intracellular neutral red was extracted using 1 M HCl in 30% MeOH. The A550 was measured using a microplate reader.

### 4.10. Effect of Botryococcene on IL-1α and PGE2 Production in UV-B-Induced Inflammation

Botryococcene-treated cells were rinsed with PBS, which was replaced with fresh HBSS. Thereafter, the cells were irradiated with UV-B (280–320 nm, 20 mJ/cm^2^) and incubated in fresh KB2 medium for 24 h. The irradiation intensity was based on the following reference [42,43]. Subsequently, IL-1α and PGE2 levels in culture supernatants were measured using ELISA kits. The cells were then lysed in 50 μL PBS containing 0.5% Triton X-100 and transferred to a 96-well plate. The BCA kit was used to estimate the total cell protein content in the cell lysates.

### 4.11. Microsphere-Based Phagocytosis Assay

Botryococcene-treated cells were rinsed with PBS, which was replaced with fresh HBSS. The experimental method was based on the following reference [42]. The cells were then irradiated with UV-B (280–320 nm, 20 mJ/cm^2^) and incubated in fresh KB2 medium for 24 h. The cells were then cultured in KB2 medium containing FluoSpheres solution (bead size 0.2 µm) for 4 h and lysed in 50 μL PBS containing 0.5% Triton X-100 and transferred to a 96-well plate. The fluorescence emission of the lysates was detected using a fluorescence reader at excitation/emission wavelengths of 535 nm/590 nm. The BCA kit was used to estimate total cell protein.

### 4.12. Measurement of Type I Collagen and MMP-1

NHEKs and NHDFs were used in this study. NHEKs were cultured at a density of 2.5 × 10^4^ cells/well in 96-well plates using KG2 medium for 24 h. The culture supernatant was aspirated and replaced with KB2 medium containing 0, 25, 50, 100, and 200 µM botryococcene for 24 h. The cells were then rinsed with PBS and incubated with HBSS. Thereafter, the cells were irradiated with UV-B (280–320 nm, 20 mJ/cm^2^) and incubated in fresh KB2 medium for 24 h, and the supernatant was aspirated. NHDFs were also cultured in 96-well plates at a density of 2.0 × 10^4^ cells/well in Dulbecco’s modified Eagle’s medium for 24 h. After incubation, the culture supernatant from the botryococcene-treated NHEK cultures was used to culture NDHFs for 24 h. The supernatants from the NHDF cultures were used to analyze type I collagen and MMP-1 production using ELISA. The cells were then lysed in 50 μL PBS containing 0.5% Triton X-100 and transferred to a 96-well plate. The BCA kit was used to estimate total cell protein.

### 4.13. Statistical Analysis

Data are expressed as the means ± SD and were assessed using analysis of variance. The Kolmogorov-Smirnov test was used to test the normality and the F-test was used to test the homoscedasticity. Individual groups were compared using Tukey’s post hoc test, with *p* < 0.05 considered as statistically significant.

## 5. Conclusions

This study demonstrates that botryococcene can be involved in the suppressing process of UV-B-induced photoaging of skin by scavenging intracellular ROS, probably via the induction of antioxidant enzymes, reducing the expression of IL-1α to alleviate cell toxicity, inhibiting melanosome accumulation, thereby down-regulating MMP-1 to promote collagen production. Botryococcene has anti-photoaging effects, including the prevention of wrinkles and blemishes on the skin, which are useful in cosmetics.

## Data Availability

Data is contained within the article or Supplementary Material.

## Supplementary Materials

The following supporting information can be downloaded at: https://www.mdpi.com/article/10.3390/md24020057/s1, Figure S1. Representative western blotting images of manganese superoxide dismutase (MnSOD) (A), glutathione 11 peroxidase (GPx), and catalase (C); Figure S2. Original Western blots images.

## References

[1] van der Rhee H.J., de Vries E., Coebergh J.W. (2016). Regular Sun Exposure Benefits Health. Med. Hypotheses.

[2] Fuller B., Smith D., Howerton A., Kern D. (2006). Anti-Inflammatory Effects of Coq10 and Colorless Carotenoids. J. Cosmet. Dermatol..

[3] Cui B., Wang Y., Jin J., Yang Z., Guo R., Li X., Yang L., Li Z. (2022). Resveratrol Treats Uvb-Induced Photoaging by Anti-Mmp Expression, through Anti-Inflammatory, Antioxidant, and Antiapoptotic Properties, and Treats Photoaging by Upregulating Vegf-B Expression. Oxid. Med. Cell Longev..

[4] Gu Y., Han J., Jiang C., Zhang Y. (2020). Biomarkers, Oxidative Stress and Autophagy in Skin Aging. Ageing Res. Rev..

[5] Endo K., Mizutani T., Okano Y., Masaki H. (2019). A Red Pumpkin Seed Extract Reduces Melanosome Transfer to Keratinocytes by Activation of Nrf2 Signaling. J. Cosmet. Dermatol..

[6] Liu F., Qu L., Li H., He J., Wang L., Fang Y., Yan X., Yang Q., Peng B., Wu W. (2022). Advances in Biomedical Functions of Natural Whitening Substances in the Treatment of Skin Pigmentation Diseases. Pharmaceutics.

[7] Seiberg M. (2001). Keratinocyte-Melanocyte Interactions During Melanosome Transfer. Pigment. Cell Res..

[8] Imokawa G., Ishida K. (2014). Inhibitors of Intracellular Signaling Pathways That Lead to Stimulated Epidermal Pigmentation: Perspective of Anti-Pigmenting Agents. Int. J. Mol. Sci..

[9] Tanaka Y., Uchi H., Hashimoto-Hachiya A., Furue M. (2018). Tryptophan Photoproduct Ficz Upregulates Il1a, Il1b, and Il6 Expression Via Oxidative Stress in Keratinocytes. Oxid. Med. Cell Longev..

[10] Kim J.Y., Shin J.Y., Kim M.R., Hann S.K., Oh S.H. (2012). Sirna-Mediated Knock-Down of Cox-2 in Melanocytes Suppresses Melanogenesis. Exp. Dermatol..

[11] Talwar H.S., Griffiths C.E., Fisher G.J., Hamilton T.A., Voorhees J.J. (1995). Reduced Type I and Type Iii Procollagens in Photodamaged Adult Human Skin. J. Investig. Dermatol..

[12] Fisher G.J., Datta S., Wang Z., Li X.Y., Quan T., Chung J.H., Kang S., Voorhees J.J. (2000). C-Jun-Dependent Inhibition of Cutaneous Procollagen Transcription Following Ultraviolet Irradiation Is Reversed by All-Trans Retinoic Acid. J. Clin. Investig..

[13] Quan T., Qin Z., Xu Y., He T., Kang S., Voorhees J.J., Fisher G.J. (2010). Ultraviolet Irradiation Induces Cyr61/Ccn1, a Mediator of Collagen Homeostasis, through Activation of Transcription Factor Ap-1 in Human Skin Fibroblasts. J. Investig. Dermatol..

[14] Moon H.J., Lee S.H., Ku M.J., Yu B.C., Jeon M.J., Jeong S.H., Stonik V.A., Zvyagintseva T.N., Ermakova S.P., Lee Y.H. (2009). Fucoidan Inhibits Uvb-Induced Mmp-1 Promoter Expression and Down Regulation of Type I Procollagen Synthesis in Human Skin Fibroblasts. Eur. J. Dermatol..

[15] Kawachi M., Tanoi T., Demura M., Kaya K., Watanabe M.M. (2012). Relationship between Hydrocarbons and Molecular Phylogeny of *Botryococcus braunii*. Algal Res..

[16] Dayananda C., Sarada R., Rani M.U., Shamala T.R., Ravishankar G.A. (2007). Autotrophic Cultivation of *Botryococcus braunii* for the Production of Hydrocarbons and Exopolysaccharides in Various Media. Biomass Bioenergy.

[17] Banerjee A., Sharma R., Chisti Y., Banerjee U.C. (2002). *Botryococcus braunii*: A Renewable Source of Hydrocarbons and Other Chemicals. Crit. Rev. Biotechnol..

[18] Niehaus T.D., Okada S., Devarenne T.P., Watt D.S., Sviripa V., Chappell J. (2011). Identification of Unique Mechanisms for Triterpene Biosynthesis in *Botryococcus braunii*. Proc. Natl. Acad. Sci. USA.

[19] Reddy L.H., Couvreur P. (2009). Squalene: A Natural Triterpene for Use in Disease Management and Therapy. Adv. Drug Deliv. Rev..

[20] Rosales-Garcia T., Jimenez-Martinez C., Davila-Ortiz G. (2017). Squalene Extraction: Biological Sources and Extraction Methods. Int. J. Environ. Agric. Biotechnol..

[21] Huang Z.R., Lin Y.K., Fang J.Y. (2009). Biological and Pharmacological Activities of Squalene and Related Compounds: Potential Uses in Cosmetic Dermatology. Molecules.

[22] Kim S.K., Karadeniz F. (2012). Biological Importance and Applications of Squalene and Squalane. Adv. Food Nutr. Res..

[23] Micera M., Botto A., Geddo F., Antoniotti S., Bertea C.M., Levi R., Gallo M.P., Querio G. (2020). Squalene: More Than a Step toward Sterols. Antioxidants.

[24] Du X., Ma X., Gao Y. (2023). The Physiological Function of Squalene and Its Application Prospects in Animal Husbandry. Front. Vet. Sci..

[25] Wolosik K., Chalecka M., Gasiewska G., Palka J., Surazynski A. (2025). Squalane as a Promising Agent Protecting Uv-Induced Inhibition of Collagen Biosynthesis and Wound Healing in Human Dermal Fibroblast. Molecules.

[26] Hillen L.W., Pollard G., Wake L.V., White N. (1982). Hydrocracking of the Oils of *Botryococcus braunii* to Transport Fuels. Biotechnol. Bioeng..

[27] Hirano K., Hara T., Ardianor, Nugroho R.A., Segah H., Takayama N., Sulmin G., Komai Y., Okada S., Kawamura K. (2019). Detection of the Oil-Producing Microalga *Botryococcus braunii* in Natural Freshwater Environments by Targeting the Hydrocarbon Biosynthesis Gene Ssl-3. Sci. Rep..

[28] Kawashima H., Umezawa M., Kijima M. (2018). Site-Selective Hydrosilylation of Botryococcene—The Algal Biomass Hydrocarbon Oil. ChemistrySelect.

[29] Kawashima H., Kijima M. (2018). Selective Synthesis of Botryococcene Pentaepoxide—The Chemical Modifications of the Algal Biomass Oil. ChemistrySelect.

[30] Oishi S., Oi K., Kuwabara J., Omoda R., Aihara Y., Fukuda T., Takahashi T., Choi J.-C., Watanabe M., Kanbara T. (2019). Synthesis and Characterization of Sulfur-Based Polymers from Elemental Sulfur and Algae Oil. ACS Appl. Polym. Mater..

[31] Sethi A., Kaur T., Malhotra S., Gambhir M. (2016). Moisturizers: The Slippery Road. Indian J. Dermatol..

[32] Rosales-Mendoza S. (2016). Algae-Based Biopharmaceuticals.

[33] Sasaki K., Othman M.B., Demura M., Watanabe M., Isoda H. (2017). Modulation of Neurogenesis through the Promotion of Energy Production Activity Is Behind the Antidepressant-Like Effect of Colonial Green Alga, *Botryococcus braunii*. Front. Physiol..

[34] Iwahashi H., Kawashima Y., Masaki H. (2020). Interleukin-1 Alpha Derived from Ultraviolet B-Exposed Keratinocytes Is Associated with a Decrease of Endocytic Collagen Receptor Endo180. Photodermatol. Photoimmunol. Photomed..

[35] Katsuyama Y., Masaki H. (2021). In Vitro and In Vivo Assessments of Phytocosmetics in Skin Care.

[36] Lee J.H., Park J., Shin D.W. (2022). The Molecular Mechanism of Polyphenols with Anti-Aging Activity in Aged Human Dermal Fibroblasts. Molecules.

[37] Frenay J., Bellaye P.S., Oudot A., Helbling A., Petitot C., Ferrand C., Collin B., Dias A.M.M. (2022). Il-1rap, a Key Therapeutic Target in Cancer. Int. J. Mol. Sci..

[38] Liu H.M., Cheng M.Y., Xun M.H., Zhao Z.W., Zhang Y., Tang W., Cheng J., Ni J., Wang W. (2023). Possible Mechanisms of Oxidative Stress-Induced Skin Cellular Senescence, Inflammation, and Cancer and the Therapeutic Potential of Plant Polyphenols. Int. J. Mol. Sci..

[39] Wei M., He X., Liu N., Deng H. (2024). Role of Reactive Oxygen Species in Ultraviolet-Induced Photodamage of the Skin. Cell Div..

[40] Oowada S., Endo N., Kameya H., Shimmei M., Kotake Y. (2012). Multiple Free-Radical Scavenging Capacity in Serum. J. Clin. Biochem. Nutr..

[41] Kurokawa H., Ito H., Matsui H. (2017). Monascus Purpureus Induced Apoptosis on Gastric Cancer Cell by Scavenging Mitochondrial Reactive Oxygen Species. J. Clin. Biochem. Nutr..

[42] Katsuyama Y., Sato Y., Okano Y., Masaki H. (2021). Intracellular Oxidative Stress Induced by Calcium Influx Initiates the Activation of Phagocytosis in Keratinocytes Accumulating at S-Phase of the Cell Cycle after Uvb Irradiation. J. Dermatol. Sci..

[43] Liu L., Xie H., Chen X., Shi W., Xiao X., Lei D., Li J. (2012). Differential Response of Normal Human Epidermal Keratinocytes and Hacat Cells to Hydrogen Peroxide-Induced Oxidative Stress. Clin. Exp. Dermatol..

