# Supplementary material for: Botryococcene Inhibits UV-B-Induced Photoaging by Scavenging Intracellular Reactive Oxygen Species
Source: Mar Drugs. 2026 Jan 30;24(2):57. doi: 10.3390/md24020057 (PMC12942113; doi:10.3390/md24020057)
Supplement: Supplementary file 1 [file marinedrugs-24-00057-s001.zip › marinedrugs-4073267-supplementary.pdf]

marine drugs

Article

Botryococcene Inhibits UV-B-Induced Photoaging by Scavenging Intracellular Reactive Oxygen Species

Hiromi Kurokawa [1,2,*] and Makoto M. Watanabe [1]

[1] Phycochemy Corporation, 4-19-1 Midorigahara, Tsukuba 300-2646, Ibaraki, Japan; m-watanabe@phycochemy.jp

[2] Faculty of Medicine, University of Tsukuba, 1-1-1 Tennodai, Tsukuba 305-8575, Ibaraki, Japan

* Correspondence: hkurokawa.tt@md.tsukuba.ac.jp

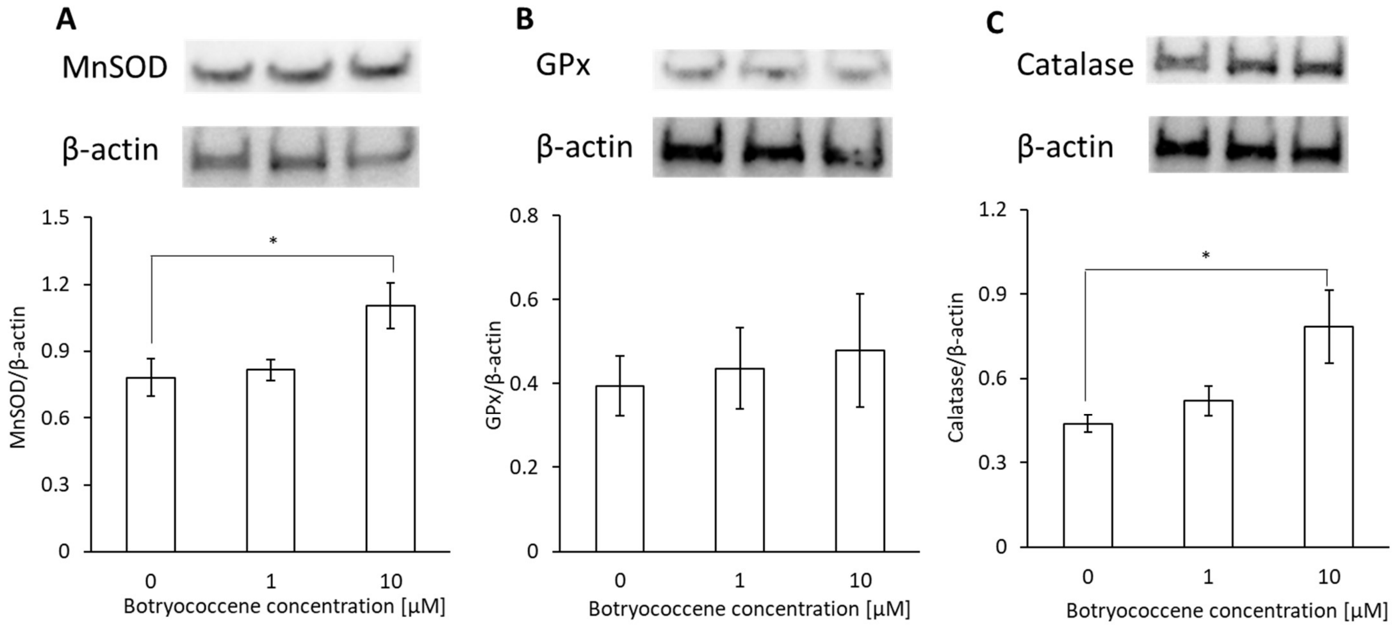

Figure S1. Representative western blotting images of manganese superoxide dismutase (MnSOD) (A), glutathione peroxidase (GPx), and catalase (C). Cells were stimulated with various concentrations (0, 1, and 10μM) of botryococcene for 24 h. The cells were lysed with the cell lysis buffer, and proteins were extracted. Protein samples were mixed with NuPAGE LDS Sample buffer (Life Technologies Japan, Ltd.) and then heated at 90°C for 10 min. The denatured protein samples were applied to the wells of 12% gels (Bio Craft, Tokyo, Japan) and subjected to SDS-polyacrylamide gel electrophoresis at 100 V for 60 min. The proteins were transferred onto polyvinylidene difluoride (PVDF) membranes (Millipore Co., Burlington, MA) by electrophoretic transfer at 2.0 mA/cm^2 for 60 min. The membranes were blocked with 15 mL of PVDF Blocking Reagent for Can Get Signal (Toyobo Co. Ltd., Osaka, Japan) for 1 h, and then treated overnight with Can Get Signal Immunoreaction Enhancer Solution 1 (Toyobo Co. Ltd.) containing anti-MnSOD, anti-GPx, or anti-catalase primary antibody at 4°C. The membranes were then washed three times with 15 mL PBS containing 0.1% (v/v) Tween-20 (PBS-T) and subsequently incubated with horseradish peroxidase (HRP)-linked anti-rabbit IgG antibody prepared in Can Get Signal Immunoreaction Enhancer Solution 2 (Toyobo Co. Ltd.) for 60 min at 25°C. Lumina Forte Western HRP Substrate (EMD Millipore, Burlington, MA) was used to develop the blots and images were captured using the Fusion FX7 Edge imaging system (Vilber Lourmat, Co, France). β-actin was also detected as a control for protein loading. Relative expression was estimated and shown in graphs. Error bars indicate SD (n = 3). *$p < 0.05$.

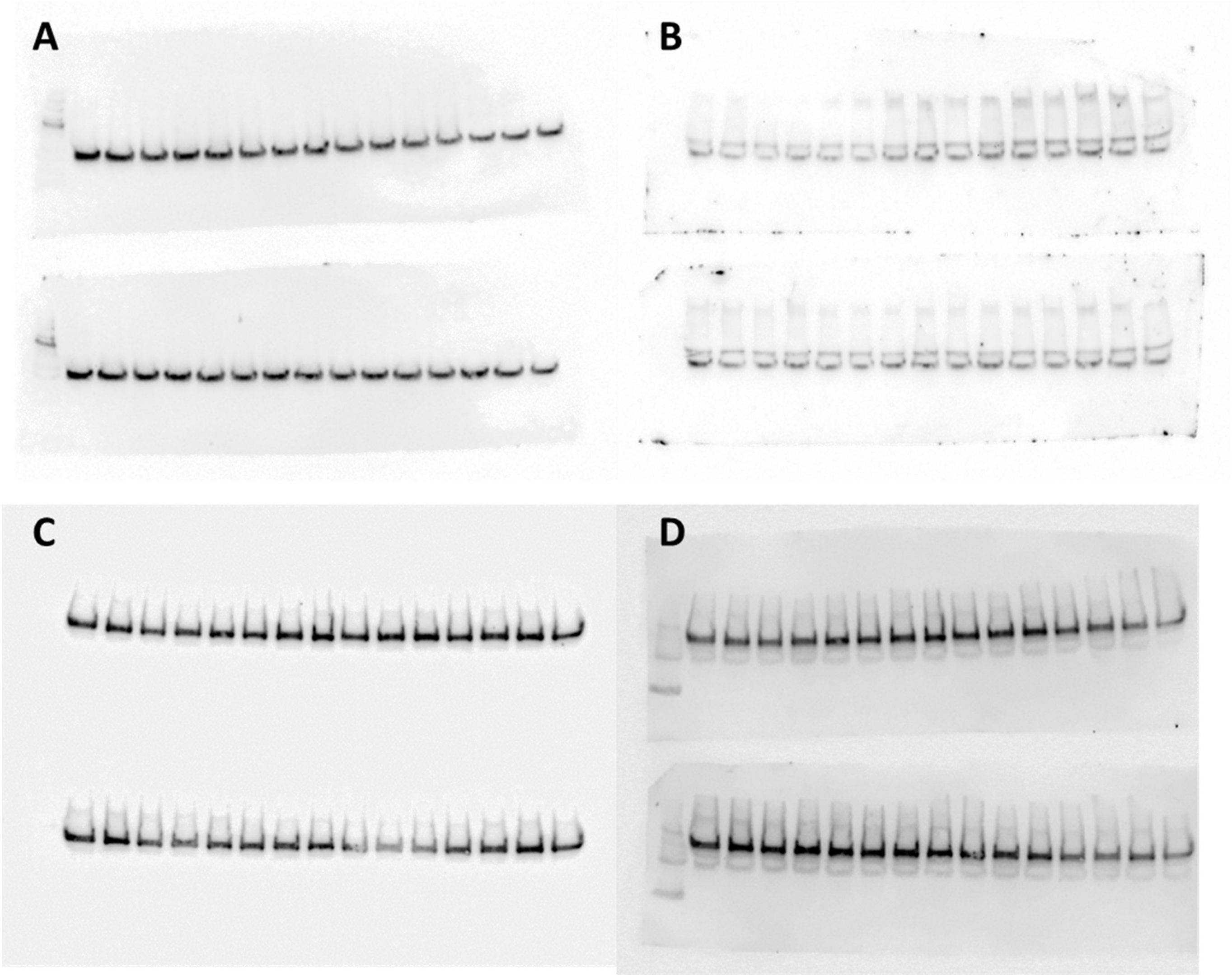

Figure S2. Original Western blots images. A: MnSOD, B: GPx, C: Catalase, D: β-actin.
